# Chronic Pelvic Pain in Endometriosis: An Overview

**DOI:** 10.4021/jocmr1288w

**Published:** 2013-04-23

**Authors:** Onofrio Triolo, Antonio Simone Laganà, Emanuele Sturlese

**Affiliations:** a Department of Pediatric, Gynecological, Microbiological and Biomedical Sciences, University of Messina - Messina, Italy

**Keywords:** Endometriosis, Chronic pelvic pain, Diagnosis, Medical treatment, Surgical treatment

## Abstract

Chronic pelvic pain (CPP) could be considered nowadays a deep health problem that challenges physicians all over the world. This because its aetiology is still unclear, the course of the disease could vary a lot among different patients and through time in the same patient, and the response to treatments is not every time successful. Among women who underwent laparoscopy for CPP, endometriosis is found in about 1/3 of the cases, while only 25% of women with histological confirmed endometriosis are asymptomatic. A wide range of variables may exert their influence on the resulting pain syndrome in endometriosis; for example, score according to American society for reproductive medicine (rASRM), size of the sub-peritoneal and pelvic wall implants, Douglas obliteration, previous surgery. It is widely accepted nowadays that central nervous system (CNS) and peripheral nervous system (PNS) seems to influence each other and this interconnection play a key role in pain modulation. Moreover, the phenomena induced by endometriosis in the pelvis, including the breakdown of peritoneal homeostasis and the induction of the production of proinflammatory and proangiogenic cytokines, are responsible of altered innervations and modulation of pain pathways in these patients. There are many proposed medical and surgical approach to treat this painful syndrome, although there is necessity of more efforts to create new non-invasive strategies that set a more accurate diagnosis of the causes of endometriotic-related CPP, and therefore facilitate its eradication.

## Introduction

Chronic pelvic pain (CPP) could be considered nowadays a deep health problem that challenges physicians all over the world. This because its aetiology is still unclear, the course of the disease could vary a lot among different patients and through time in the same patient, and the response to treatments is not every time successful. So, actually the world medical literatures suggest to engage a multidisciplinary approach to this syndrome [1]: in fact, it is needed a clinical integrated know-how about pelvic organs anatomy and physiology as well as about pathology, that could include neurological and/or musculoskeletal problems. Some other patients could have an aetiology derived from psychiatric diseases that vent their devastating symptoms on the pelvic area, from drug addiction or rebound phenomena to the drug use. CPP represent also a public financial problem, because it deeply increases medical cost for diagnosis and therapy of this type of patients: just considering USA, it costs approximately $881.5 million per year [2]. According to Gelbaya and El-Halwagy [3], CPP is cause for approximately 40% of laparoscopies and 10% to 15% of hysterectomies. It is also an annoying disease that affect deeply and negatively woman’s quality of life: this considering also that CPP is often associated to migraine and headache, regardless if CPP is related or not to endometriosis [4]. Leserman et al [5], underlined that patients with diffuse abdominal/pelvic pain had more trauma and worse mental and physical health status compared with patients with vulvovaginal pain and cyclic pain, and also had poorer health than patients with neuropathic and fibroid pain. This disease could be defined as the presence of non-cyclic pain of 6 months duration or longer that localizes to the anatomic pelvis and is severe enough to cause functional disability and require medical or surgical treatment [6]. One of the consensuses about it is that we can make diagnosis of CPP when the pain started from the pelvic area and remain for at least 3.6 months. CPP aetiology could be related to different situations, and often it derived from more than one of the following [7]: 1). Gynaecological and obstetric: post-surgical pain due to the presence of adhesions that can involve pelvic organs and walls; chronic cervical infection for cervical stenosis; post-surgical complication after cryo/laser/diathermy surgery for portio diseases; pelvic inflammatory disease (PID); endometriosis and adenomyosis; 2). Urologic: recurrent and/or interstitial cystitis; complication after urologic surgery; nephrolithiasis; urolithiasis; 3). Gastrointestinal: irritable bowel syndrome; chronic inflammatory bowel disease, diverticulosis, polyposis; 4). Vascular disease: pain is thought to arise from dilated pelvic veins in which blood flow is markedly reduced (pelvic congestion syndrome) [8]; 5). Musculoskeletal disease; 6). Neurological: altered spinal cord and brain processing of stimuli in women with chronic pelvic pain [8]; 7). Psychological: it is wide reported that psychological diseases, such as depression and/or anxiety disorder could vent on the pelvic area [9].

According to Neis et al [1], in nearly 1/3 of the cases the reason for the pain is an endometriosis and in another third, adhesions are responsible for the pain. Other authors stated that there are four of more common disorders associated with chronic pelvic pain (endometriosis, adhesions, irritable bowel syndrome, and interstitial cystitis) [10]. Mathias et al [2], reported that among 5,263 U.S. women, 773 (14.7%) had chronic pelvic pain within the past 3 months, and that in 61% of the cases the aetiology was unknown. They underlined also that women diagnosed with endometriosis reported the most health distress, pain during or after intercourse, and interference with activities because of pain. Howard [10] estimated CPP prevalence in 3.8% in women of all ages. Other authors [11], referring to a clinical population of reproductive-age women, found that reported prevalence of dysmenorrhoea, dyspareunia, pelvic pain, and irritable bowel syndrome was 90, 46, 39, and 12%, respectively. Moreover, they remark that African-American race was found to be a risk factor for pelvic pain, but it was not associated with age, parity, marital status or education. The aetiology of CPP may arise from multiple sites in the pelvis including the bladder, pelvic peritoneum, and vulva: Stanford et al [12], collected 64 patients undergoing intravesical potassium sensitivity test (PST), cystoscopy with double-fill hydrodistension, a physical examination for vulvar pain, pelvic pain/urgency/frequency (PUF) screening questionnaire and laparoscopy to assess the presence of peritoneal pathology. They found that bladder pain, peritoneal pathology, and vulvar pain are independent risk factors of CPP although a trend of severity was noted in patients who had worse symptoms (increased voids per day, urgency, pain, and PUF scores). One of the most important things approaching CPP is to take detailed patient history, focusing mainly of reproductive, gastrointestinal, musculoskeletal, urologic, and neuropsychiatric symptoms and signs. It also really important to ask patient’s previous examination and diagnostic process, in order to avoid unnecessary and sometimes invasive repeating of them (first for the patient and second for the medical costs). Physicians have to check the pattern of CPP [6], including: 1). Pain location and distribution; 2). Pain duration; 3). Factors that can provoke or intensify pain; 4). Factors that can abolish or decrease pain; 5). Type of pain; 6). Intensity of pain; in this analysis it could be better to use standard rating system (pain scales).

## Chronic Pelvic Pain and Endometriosis

Endometriosis is a disorder characterised by the ectopic presence and growth of functional endometrial tissue, glands and stroma, outside the uterus [13]. Endometriotic foci can be found anywhere in the pelvis, including the peritoneal surface of endopelvic structures and the ovaries. Endometriosis is classified depending on the number, size and superficial and/or deep location of endometrial implants, plaques, endometriomas and/or adhesions, as follows: stage I (minimal, 1 - 5 points), stage II (mild, 6 - 15 points), stage III (moderate, 16 - 40 points) and stage IV (severe > 40 points) following the revised American Society for Reproductive Medicine classification for Endometriosis (American Society for Reproductive Medicine, 1996) [14]. Among women who underwent laparoscopy for CPP, endometriosis is found in about 1/3 of the cases, whereas among who had not CPP endometriosis is found in no more of 5% [15]. Similar finding are reported among adolescent girls with CPP not responding to conventional therapy: Laufer et al [16], in fact, found that more than two thirds of their study population (69.6%) had endometriosis at laparoscopy, staged at I or II according to the American Fertility Society’s classification system. Fauconnier et al [17] found that endometriosis appears to be responsible for chronic pelvic pain symptoms in more than half of histologically confirmed cases. Another important element to assess the strong correlation between endometriosis and CPP is representing by data of Randall et al [18]: they enlighten that high serum level of antiendometrial antibody (AEA) in women with CPP could screen patients suspected of having endometriosis. Considering that a local increase in estrogens levels is characteristic of patients with ovarian endometrioma lesions, and that in any case estrogens could promote endometriotic cells proliferation [19], also affecting the increase in the immune suppressive potential of Treg cells [20], Berkley et al [21] suggest that mechanisms underlying CPP and sensitivity to estrogens involve the growth into the ectopic endometrial tissue of a nerve supply, which could have a varied and widespread influence on the activity of neurons throughout the central nervous system (CNS). It also very important for the physician to understand if CPP is caused by endometriosis, or endometriosis and CPP are separated entity. According to Chapron [22] CPP is related in the most of cases to deep infiltrating pelvic endometriosis, and is dominated by dyspareunia and pain that recur according to the menstrual cycle. This deep endometriosis could affect frequently utero-sacral ligaments. A wide range of variables may exert they influence on the resulting pain syndrome in endometriosis; for example, score according to American society for reproductive medicine (rASRM), size of the sub-peritoneal and pelvic wall implants, Douglas obliteration, previous surgery (numbers and type of procedures). Considering that pain attributed to endometriosis occur in women without endometriosis and pain and severity correlate poorly with lesion characteristics, it is widely accepted nowadays that the experience of pain is due to activity in the CNS [23]. Although the most important symptom in CPP in endometriosis is of course pain, it could be expressed in a wide range combination of type, such as dysmenorrhea, dyspareunia, dysuria, dyschezia, non-menstrual chronic pelvic-abdominal muscle pain [23]. Dysmenorrhea is independent of the macroscopic type of the lesions or their anatomical locations and may be related to recurrent cyclic microbleeding in the implants [17].

## Aetiopathogenetic Mechanisms of Endometriosis-Associated CPP

Like is well evidenced by Howard [15] and Vercellini et al [10], pain may be due to nociceptive, inflammatory, or neuropathic mechanisms, and probably all three of these mechanisms are relevant to endometriosis-associated pelvic pain. In an extensive work, Berkley [21] enroll many interesting finding on CPP arising mechanism based on murine model. First of all he remark that the female reproductive organ are innervated by pelvic (for vagina and cervix) and by hypogastric nerve (for cervix and uterine horn), and that nociceptive stimuli could be exacerbate or decrease by reproductive (and consequently hormonal) status for the patient. So central nervous system (CNS) and peripheral nervous system (PNS) seems to influence each other, because CNS neurons responsive to stimulation of the reproductive tract also respond to stimulation of skin and other internal organs. Since this, it was observed a dynamic interconnection of different stimuli entering the CNS via gateways through the spinal cord, dorsal column nuclei, and solitary nucleus. Another important element to consider is that pathophysiology in one organ can influence physiology and responses to pathophysiology in other organs, because CNS is organized by intensive cross-system and viscero-visceral interaction. Focusing on endometriosis, this means that CPP could arise and be exacerbated also by other organ’s condition. The importance of CNS in influencing pain experience is also underlined by work of As-Sanie et al [24]: they applied voxel-based morphometry to determine whether women with CPP with and without endometriosis display changes in brain morphology in regions known to be involved in pain processing, and they found that women with CPP with or without endometriosis displayed decreased gray matter volume in brain regions involved in pain perception, including the left thalamus, left cingulate gyrus, right putamen, and right insula. The first and most important element to consider in order to understand endometriosis-related CPP is that pain depend non only by endometriotic foci innervation. In fact, it could arise through different pathways: one of the clearest reason is that endometriotic lesion could provoke a compression or infiltration of nerves [25]. Other possible causes of arising CPP could be represented by increase of nerve growth factor (NGF) in the endometriotic lesion area, and this may worse the disease progression by two different way: on one hand it could emprove “neural sprouting” and so create new painful afferents to the CNS, and, on the other hand, it could be considered itself a mediator that may exacerbate the CPP [26]. Another cell mediator, the vascular endothelial growth factor (VEGF), could contribute to neoangiogenesis of the nerve vessels (in order to support “neural sprouting”), and so could enhance CPP [27]. This is incongruent with the finding of Garcia-Manero et al [28] that evidenced that pain symptoms in ovarian endometriosis are not correlated with VEGF serum levels and VEGF cellular expression, but probably with microvascular density (MVD). Other authors [29] state the importance of neoangiogenesis in the development of endometriosis, and the possible role of anti-angiogenic therapy: they use a murine model of endometriosis and administered an adenovirus vector to overexpress the gene for a natural angiogenesis inhibitor angiostatin, and found that the disease was eradicated in all endometriosis-induced mouse. Berkley et al [21] underlined that endometriotic lesions could stimulate the growth of their own innervations by sensory and sympathetic fibers. CPP itself (related or not to endometriosis) seems to be associated with proliferation of small-diameter nerve fibers through the myometrial stroma of uterus, with an pattern of proliferation in some cases [30]. There is also a recent work, realized by Poli-Neto et al [31], that investigate about expression of capsaicin receptor (transient receptor potential vanilloid type-1 (TRPV1)) in the peritoneum of women with CPP. Using immunochemical analysis, they found that TRPV1 immunoreactivity was detected in the nervous tissue and epithelium of endometriotic lesions, and that this immunoreactivity was greater in women with CPP respect to controls. This finding suggest that endometriotic lesions could enhance CPP through TRVP1 nociception pathways. Interestingly, some authors [32] found that this innervations is denser in deeply infiltrating endometriosis (DIE) lesions respect to other lesion type (and this could make relation with the fact that DIE lesions are frequently associated to CPP respect to other lesions type, see after). Other authors [33], on the contrary, state that there are no differences in detection of peritoneal “neural sprouting” (using immunocytochemistry staining with an antibody to neurofilament) between endometriotic patients and controls. However, they found that endometriotic lesions have more lymphocytic infiltration and mesothelial hyperplasia respect to controls: we could hypothesize that this finding is consistent with the fact that infiltration of lymphocytes contributes to local inflammation and to release of cytokines that can worsen chronic pelvic pain [34]. About breaking peritoneal fluid homeostasis, there is evidence that leptin [35] and 6-keto-prostaglandin F1 alpha (6-KF) [36] are significantly elevated in peritoneal fluid of women with endometriosis, and this may play a role in endometriosis-associated pain. Moreover, Drosdzol-Cop et al [37], found that adolescents with endometriosis had significantly higher concentrations of serum and peritoneal fluid IL-4, lower peritoneal fluid IL-2, higher peritoneal fluid levels of IL-6, TNF-α and glycodelin A compared to controls: these findings could represent also markers of peritoneal inflammation that contribute to CPP.

## Diagnosis

Considering that CPP is related in most of cases to deep infiltrating endometriosis, it seems to be really important to state, as clinically as with diagnostic imaging, location, size and depth of the implants, in order to address surgical procedure at target. So, some authors suggest using magnetic resonance imaging to assess the pre-operative point [22]. The “gold standard” to assess the presence of endometriotic lesions in CPP is actually considered laparoscopy [38], although many authors [3, 24] state that investigation by laparoscopy often reveals no obvious cause for pain: laparoscopic view, in fact, can accurately state the location and extent of lesions, but it does not have the same precision in quantify their depth. On the contrary, the “Consensus statement for the management of chronic pelvic pain and endometriosis” [39] assess that for women in which endometriosis is the suspected cause of the pain, laparoscopic confirmation of the diagnosis is unnecessary. About this, Newham et al [40] found that there is no differences in age, parity, duration of pain, frequency of dysmenorrhoea and dyspareunia or the presence of gastrointestinal or urinary symptoms between patient with laparoscopic no obvious cause for CPP and patients in which cause of CPP was identified. Moreover, although laparoscopy is the best way to individuate endometriotic lesions, sometimes the tissue is not histologically classified as endometriotic, especially in the minimal and mild stage [23]. Momoeda et al [41] analyzed 1,092 women with endometriosis, dividing them in two group infertility (infertility group; n = 476) or pain (pain group; n = 616): they found that the frequencies of chronic pelvic pain and dyspareunia increased with disease stage either in the infertility group, in the pain group or in the aggregate. Moreover, they found that a parallel increase in the severity of dysmenorrhea with disease stage was observed in the infertility group, but not in the pain group. However, laparoscopy is fundamental, since there is evidence that other kind of diagnostic tools, such as pelvic ultrasound and bimanual pelvic examination often fails to individuated the cause (or causes) of CPP, especially when it is endometriosis-related [42]. Another important thing to consider is that histological confirmation of endometriotic tissue is not certainly related to CPP. Moreover, many authors [6, 22, 43, 44] evidenced a poor correlation between severity of the endometriosis (stated according to rASRM or R-AFS) and level of CPP, although there is evidence that advanced endometriosis is more frequently related to dysmenorrhea and deep dyspareunia in comparison to early disease [45]. According to Howard et al [14], the discrepancy observed between endometriosis stage and severity of CPP may be due to variable roles of different endometriosis-related pain mechanisms, and, in our opinion, also to the different influence that each mechanism have on the others. Vercellini et al [43] evidenced that the frequency and severity of deep dyspareunia and the frequency of dysmenorrhea were less in patients with only ovarian endometriosis than in those with lesions at other sites, and that the severity of deep dyspareunia was related inversely to the endometriosis score. Hurd suggests to use three criteria to assess that CPP in related to endometriosis: first, is that pelvic pain should be cyclic because endometriosis is a hormonally responsive disease. The second thing to consider is that endometriosis should be diagnosed surgically to avoid overdiagnosing of this condition, and the third is that medical or surgical treatment of endometriosis should result in prolonged pain relief [46].

Exerting a gross classification, endometriotic lesions could be divided into superficial peritoneal endometriosis, deeply infiltrating endometriosis (DIE) and ovarian (cystic) endometriosis [47]. According to Faucconier et al [44], only DIE lesions are associated with CPP, especially when this type of lesions involve precise anatomical locations (severe deep dyspareunia, painful defecation) or organs (functional urinary tract signs, bowel signs) [48]. Moreover, his research team state that the frequency of severe dysmenorrhea increased with Douglas pouch endometriotic adhesions and decreased with parity, that the frequency of noncyclic chronic pelvic pain is higher when DIE involve the bowel, and finally that gastrointestinal symptoms are associated with bowel or vaginal DIE locations [48]. DIE-related pain seems to be in relation with compression or infiltration of nerves in the subperitoneal pelvic space by the implants [17]. This evidence seems to be confirmed by Dai et al [49]: they collected data from 354 patients, including 177 patients with DIE lesions and 177 without. Among DIE lesions, 60.7% of the uterosacral ligament nodules were bilateral, 44.6% of the cul-de-sacs were completely blocked and in in 19.9% there was rectum invasion. They found that the duration of pain suffering in DIE patients was much longer than that of non-DIE patients, and that DIE lesions were associated with severe pain symptoms. On the contrary, the correlation between CPP and endometriomas is less strong [43], although there is evidence that endometriomas is frequently associated with the presence of DIE lesions [50]. Okaro et al [51] collected a population of 120 women with CPP undergoing transvaginal ultrasonography before either diagnostic or operative laparoscopy, checking the presence of so-called “hard makers” (for example, endometrioma or hydrosalpinx) and “soft markers” (reduced ovarian mobility and site-specific pelvic tenderness): with this approach, they demonstrated the possibility of reduce the necessity of laparoscopy through the search of these ultrasound-based “soft” and “hard” markers for the prediction of pelvic pathology. Another important aspect is that often there is coexistence of interstitial cystitis and endometriosis in patients with chronic pelvic pain (“Evil Twins” syndrome): Chung et al [52], in fact, collected 178 women with CPP who presented with bladder base/anterior vaginal wall and/or uterine tenderness, with or without irritative voiding symptoms. They found presence of endometriosis in 134 (75%) patients, and of interstitial cystitis in 159 (89%) patients. So, they suggest to perform both laparoscopic and cystoscopic examinations concurrently with the patient anesthetized in the initial evaluation and treatment of CPP, in order to avoid unnecessary delay in making the diagnosis of “evil twins” syndrome. Fundamental in analyzing endometriosis-related CPP is pain assessment: actually, the most accurate studies in literature are based on a classification that divide dysmenorrhoea, dyspareunia and CPP as either absent, mild, moderate or severe, focusing also on patient’s debility [53]. Fabbri et al [54] used McGill pain questionnaire (MPQ) to get information about chronic pelvic pain associated with endometriosis: they collected 55 women undergoing laparoscopy for severe endometriosis, and administered MPQ before surgery and at the 6-month follow up. They found that postoperative index of pain intensity was < 1 in 50% of patients, > 2 in 25% of patients while 25% of patients did not experience postoperative pain, and that pain intensity significantly decreased after laparoscopic treatment. Considering this useful classification, is desirable in future to adopt additional element to consider also pain in relation to different endometriotic foci (superficial peritoneal endometriosis, DIE and endometriomas) and pain outcomes [23]. When all possible anatomical causes of CPP were excluded, is recommended to address patient to counselling or psychotherapy [8]. This because, like evidenced before, CPP cause is often unclear, and for this reason it has been argued that psychological and social factors contribute to such “unexplained” pain [7].

## Medical Treatment

Like is well evidenced by Howard [14], treatment of chronic pelvic pain may consist of two approaches. On one hand, is important to treat chronic pain considering itself as a diagnosis, and, on the other hand, to treat diseases or disorders that might be a cause of or a contributor to chronic pelvic pain. Usually, the first step in treatment of CPP (related or not to endometriosis) is represented by analgesic drugs. Among endometriotic women this medical treatment seems to be not effective, or, at least, there is no evidence that can alleviate pain in every patient. There is in literature evidence that patients with peritoneal-only endometriosis suffering from moderate or severe chronic pelvic pain have significantly more frequent COX-2 overexpression at immunohistochemical analysis. Since this, Buchweitz et al [55] suggest that this kind of patients could emprove CPP using COX-2 inhibitors. Another research group [56], moreover, evidenced that antioxidant vitamins (vitamins E and C) could reduce endometriosis-related pelvic pain and also causes a reduction of inflammatory markers in the peritoneal fluid, such as IL-6 and monocyte chemotactic protein-1 (MCP-1). Among hormonal therapy, many drugs was tested to treat endometriosis: according to many authors [57-59] combined oral contraceptives (OC, estrogens and progestins), danazol, gestrinone, medroxyprogesterone acetate and GnRH agonists have good chance to reduce endometriosis-related CPP, and have equal effectiveness to suppress ovarian production of estrogens and progesterone. In particular, Szendei et al [60] found that after laparoscopic surgery for endometriosis, the use of monophasic OC treatment could significantly reduce pain scores and the necessity of other radical operative solution. This finding is shared also by Gambone et al [39] who moreover state that for women in whom endometriosis is the suspected cause of CPP the best approach seems to use medical therapy, including second-line therapies such as danazol, GnRH agonists, and progestins until there is necessity for surgery. Although OC could be used as a treatment, Taskin et al [61] suggest another possible diagnostic role in differentiating endometriosis-related CPP from other causes: they found that unresponsiveness to low-dose OC after 4 to 6 months is highly sensitive and predictive of organic pelvic disorders, such as endometriosis as the cause of pelvic pain. Stones et al [8] reported that medroxyprogesterone acetate was associated with a reduction of pain during treatment. However, hormonal therapy does not eliminate endometrial implants, so stopping the treatment CPP (and other endometriosis related symptoms) could come back and exacerbate pre-treatment condition. The rational of using progestogens combined or not with estrogens is related to two different actions: on one hand, their anti-angiogenic, immunomodulatory and anti-inflammatory effects, and, on other hand, their action of inhibition of implantation and growth of refluxed menstrual endometrium [62]. About other kind of hormonal therapy, Leuprolide acetate (GnRH agonist) could be effective as pain reliever in women with endometriosis-associated CPP, but some authors [63] state that it could be effective also in CPP in women without endometriosis. Regirord et al [64] compared the efficacy of the GnRH-agonist leuprorelin acetate depot and of the gestagen lynestrenol, in a population of 48 women after laparoscopic surgery for endometriosis at different stage, and evidenced that the improvement in the symptoms of dysmenorrhea, chronic pelvic pain and dyspareunia was more pronounced in the group who used leuprorelin acetate respect to the other one. Some authors [65] found also that intra uterine system with levonorgesterl (LNG IUS) could be useful and decrease endometriosis-associated CPP, for the same reasons assessed before about use of progestins. When chronic treatment is indicated, Choktanasiri and Rojanasakul [66] suggest that buserelin acetate subcutaneously implants could be helpful to reduce the pain. Comparing hormonal medical treatment for endometriosis-related CPP, Brown et al [67] state that there is no evidence of a difference in objective efficacy between dydrogesterone and placebo, between depot administration of progestogens versus other treatments (low-dose oral contraceptive or leuprolide acetate), between oral progestagens over other medical treatments, and between anti-progestagens (gestrinone) compared with Danazol. Raloxifene therapy, administered after surgical excision of endometriotic implants, seems to accelerate the relapse of CPP [68]. Huber et al [69], considering that clinical evidence shows that pregnancy leads to alleviation of endometriotic symptoms such as CPP and dysmenorrhea, used human chorionic gonadotrophin (HCG) injections to treat endometriosis, and evidenced a clinically relevant reduction in pain intensity. HCG induce up-regulation of PAI2, DUSP6, PLAU and MMP1 genes in endometriotic stromal cells [70], so there is the possibility this gene cluster may influences in some way endometriosis-related CPP. However, also anti-progestin could be effective as pelvic pain reliever [58]. Since aromatase expression in endometriotic tissues seems to be exaggerated [71], it is documented in literature the use of aromatase inhibitors (associated with another drug that suppress ovarian estrogens and progestins production) to treat endometriosis-related CPP [72]. In particular, Ailawadi et al [73] evidenced that the combination of letrozole (an aromatase inhibitor) and norethindrone acetate achieved marked reduction of laparoscopically visible and histologically confirmed endometriosis in all patients in which this treatment was administered. Another novel approach to the problem is represented by use of norethisterone acetate: collecting a study population of 40 women with colorectal endometriosis, Ferrero et al [74] treated them with 2.5 mg/day of this drug, and found that this therapy may determine a relief of pain and gastrointestinal symptoms, but not in all patients. In a most recent work [75] they evidenced also that letrozole combined with norethisterone acetate is more effective in reducing pain and deep dyspareunia than norethisterone acetate alone, but for letrozole using we need to consider the high rate of adverse effect, the high cost and that it does not influence the pain recurrence. Also the use of of the association between N-Palmitoylethanolamine and transpolydatin seems to be effective in the management of endoemtriosis-related CPP after the surgery, reducing dysmenorrhoea, dyspareunia and pelvic pain [76]. In endometriosis changes occur in the peritoneal microenvironment, involving peritoneal macrophages and attracting peripheral mononuclear cells, recruited from the blood into the peritoneal cavity: these peritoneal fluid mononuclear cells (PFMCs), as well as endometriotic cells, secrete different patterns of cytokines that cause inflammation and angiogenesis [34]. Considering these finding, some authors in literature tried to use substances that could avoid or at least decrease inflammation and angiogenesis, such as inhibitor of TNF-alpha [77], of peroxisome proliferators-activated receptors gamma [78], and of angiogenesis [79]. In fact, TNF-alpha, one of the most well-know cytokine, seems to play a key-role in endometriosis inflammation [34], although Lv et al [80] found that using infliximab (an anti-TNF-alpha drug) there was no influence in reduction of endometriotic-related CPP, dysmenorrhoea and dyspareunia. Another early-studied field is represented by mTOR/AKT pathway, that is strongly related to DIE lesions: Leconte et al [81] found that mTOR/AKT inhibition by temsirolimus decreased endometriotic cell proliferation both in vitro and in vivo in a mouse model of DIE. So, considering (see before) that endometriosis-related CPP depends in most of cases by DIE lesions, mTOR/AKT pathway inhibition could represent a future way to reduce this type of symptoms.

## Surgical Therapy

Treatment of CPP in endometriosis is not yet well encoded, because of the unclear aetiology and variable response to treatments (as surgical as medical). Some authors [22] state that in CPP related to deep infiltrating endometriosis the physicians must consider as first step the surgery, with a complete exeresis of the targeted lesions, whereas medical treatment could be only palliative in the majority of cases. Among the surgical techniques, laparoscopy is efficient for bladder, utero-sacral ligaments and vaginal deeply infiltrating endometriosis, whereas for bowel endometriotic lesions there is indication to use open surgery (laparotomy). About this, Gambone et al [39] assess that when surgery is needed, the best way to reduce morbidity is laparoscopy, although they evidenced that the clinical outcomes are comparable for the laparoscopy and laparotomy. The most important concept approaching endometriosis-associated CPP is that surgery must be radical, removing all the lesions (for this is also important a good mapping with pre-operative magnetic resonance) [42]. Despite radical surgery, some authors [82] found that pain re-arise and the patients undergo reoperation in 50-60% of the cases by 5 - 7 years. There is also evidence that re-operations rates are lower after hysterectomy than after operative laparoscopy [83], probably because uterus contain a great number of neural elements that can contribute to arising and exacerbation of CPP. Moreover, treatment of DIE lesions seems to have more effectiveness in long-term pain relief [84] respect to treatment of endometriomas and superficial peritoneal endometriosis [42, 48], although Kaiser et al [85] evidenced that peritoneal endometriotic lesions could worse the pain after the first step surgery and, for this reason, suggest to pay particular attention in their excision. Surprisingly, Sutton et al [86] showed that CPP return sooner after surgery in patients with endometriosis at minimal and mild stages. The presence of thick adhesions could represent a marker of easy reforming lesions and consequently lesion-associated CPP, respect to thin adhesions [87]. Moreover, deep dyspareunia is strongly correlated with the presence of dense pelvic adhesions [45]. About this, some authors [8] reported that adhesiolysis was not associated with an improved outcome on CPP apart from where adhesions were severe. Confirming this finding, Li et al [88] analyzing 662 patients with endometrioma and pelvic adhesion undergoing laparoscopic ovarian endometrioma excision, found that endometrioma adhesion rate is related to severer pelvic pain symptoms, and that postoperative pain recurrence rate is more frequent in patients with moderate-to-severe endometrioctic adhesion. Since, like evidenced before, it seems that the most important causes of endometriosis-related CPP are DIE lesions [48], Chopin et al [89] suggest that complete surgical excision of this kind of lesions results in a statistically significant reduction in painful functional symptoms, whatever the main location of DIE lesions or preoperative characteristics of the patient. Another important thing to underline is that, among women in reproductive age, surgery timing should be setted in the woman’s follicular phase after menses, in order to avoid reimplant of endometrial debris via retrograde menstruation in healing peritoneal area [90]. Among the various surgical techniques to manage endometriosis-associated CPP, evidences about laparoscopic utero-sacral nerve ablation (LUNA) or presacral neurectomy (PNS) did not show conclusive data [8, 91]. In particular, Vercellini et al [92] suggest that presacral neurectomy and amputation of the uterosacral ligaments seems to be uneffective to treat endometriosis-related CPP and did not demonstrate better results with the use of lasers rather than electrocoagulation. The same opinion is shared by El-Din Shawki [93], who suggests that LUNA can be a last alternative option in well-selected patients for control of chronic pelvic pain without endometriosis. However, other authors [94] state that the short term results for PSN and LUNA seem to be similar, although PSN has better results in the long term. Some good results about PSN came from data of Jedrzejczak et al [95] and Zullo et al [96] who evidenced that this technique could promote long-term pain relief, significantly reducing the associated dysmenorrhea. In this field, Gambone et al [39] suggest that there is some evidence that adjuvant PNS adds benefit for CPP, but currently there is inadequate evidence to support the use of LUNA or uterine suspension. This opinion is shared also by Johnson et al [97], who found that there is a significant reduction in dysmenorrhea at 12 months follows up in women with chronic pelvic pain in the absence of endometriosis who underwent LUNA, but no significant difference in non-menstrual pelvic pain, deep dyspareunia or dyschezia. Kanazi et al [98] suggest also another surgical nerve-blocking technique to treat endometriosis-related CPP: they blocked superior hypogastric plexus (SHP) and found that all patients had significant pain relief immediately after the block, although the pain scores postblock ranged from 0 to 4/10 and the duration of pain relief varied from 1 to 14 days.

## Conclusions

Chronic pelvic pain is currently an entity that causes big discomfort for women all over the world, both from the point of view of medical management that of social costs. The diagnosis of chronic pelvic pain is very often not due to a certain cause, and, when the cause is found, often is reached after several diagnostic tests, including invasive ones. The endometriosis nowadays seems to stand out among the causes of this pain syndrome, although currently the efforts of the medical world are intended to clarify clearly and definitively the causal relationship between the two diseases. It seems, in fact, that the phenomena induced by endometriosis in the pelvis, including the breakdown of peritoneal homeostasis and the induction of the production of proinflammatory and proangiogenic cytokines, are responsible of altered innervations and modulation of pain pathways in these patients. On the other hand, there is necessity of more efforts to create new non-invasive strategies that set a more accurate diagnosis of the causes of endometriotic-related chronic pelvic pain, and therefore facilitate the pre-surgical and surgical treatment. Besides this, there is a need to implement studies related to medical and surgical therapy, with accurate meta-analysis based on large study populations.
